# Predictors of Treatments Acceptable to Patients for Late-Life Depression

**DOI:** 10.1155/2013/207493

**Published:** 2013-10-23

**Authors:** Gerald J. Jogerst, Shimin Zheng, Erik Vanderlip

**Affiliations:** ^1^Department of Family Medicine, University of Iowa Roy J. and Lucille A. Carver College of Medicine, 200 Hawkins Drive, Iowa City, IA 52242, USA; ^2^Department of Biostatistics and Epidemiology, Eastern Tennessee State University, P.O. Box 70259, Johnson City, TN 37614, USA

## Abstract

*Objectives*. Describe older patients' perceptions about depression and characteristics associated with acceptance of treatments. *Design*. Cross-sectional study. *Setting*. Three primary care clinics in Iowa. *Participants*. Consecutive sample of 529 primary care patients. *Measurements*. Depression screening tool (a 9-item patient health questionnaire [PHQ-9]) and questionnaire including sociodemographic data, patient attitudes about depression, and acceptability of different treatments. *Results*. Mean age was 71.9 years (range 60–93 years), 314 (59%) female. Among the 529 participants, 93 (17.5%) had history of depression and 60 (11.3%) had PHQ-9 scores of 10 or greater. Participants believed depression is a disease for which they would use medication and counseling. Accepting medications from primary physicians was strongly associated with a past history of depression (*P* < 0.01) and with agreeing that depression needs treatment (*P* < 0.01). Counseling was not acceptable for those believing that they can control depression on their own (*P* < 0.01). Older patients (*P* < 0.001) and those with higher education levels (*P* < 0.01) were less likely to accept herbs or supplements as treatment options. Willingness to discuss treatments with family was associated with not using alcohol as a treatment and acceptance of all other treatment options (*P* < 0.001). *Conclusions*. Attitude that depression is a disease and the willingness to discuss depression with family may enhance treatment acceptance.

## 1. Introduction

 Clinically relevant depressive symptoms are present in about 20% of community-dwelling persons aged 75 years and older [1]. Late-life depression affects at least 5–10% of older primary care patients [2]. Depression often coexists with chronic medical conditions, such as dementia, Parkinson's disease, and vascular disease, and influences the prognosis of other diseases [3–5]. Depression in primary care patients aged 55 years and older has a poor prognosis [6]. Persistent depressive symptoms are associated with lower perceived health status, patient barriers to self-management [7], and higher mortality [8]. Remission of symptoms of depression is associated with reduction in mortality [9].

About 40–50% of older adults with nonpsychotic major depressive disorder (MDD) respond satisfactorily to the first prescribed antidepressant medication [10, 11]. With vigorous and persistent treatment, up to 90 percent of older depressed patients will respond to drug therapy [12]. Within two years 60% of community-dwelling older adults with MDD became depressed again unless they were maintained on antidepressant medication [13]. Poor adherence to taking medications may account for a substantial proportion of treatment failures [14, 15]. Patients' compliance and continuous treatment are important. 

Adherence to depression treatment in late-life depression is associated with potentially modifiable factors, including patients' attitudes, beliefs, and social norms [16]. Attitudes include perceived effectiveness of treatment, preferences for types of treatment, and patient resistance to viewing depression as a medical disease [17, 18]. Social norms including stigma of the depression diagnosis and the impact of caregivers' agreement with treatment recommendations influence treatment compliance [16, 19, 20].

The purpose of this study is to describe the perceptions of older ambulatory care patients about depression and characteristics associated with acceptance of various depression treatments. 

## 2. Materials and Methods

### 2.1. Subjects

Between November 2008 and March 2009 all persons 60 years of age or older visiting one of three primary care clinics were given a packet of research materials and asked by the clinic receptionists to consider participating in the study. Two of the clinics, the Family Medicine and General Internal Medicine clinics, are located at the University of Iowa Hospitals and Clinics in Iowa City, IA. The third clinic is a free-standing primary care office in North Liberty, IA. After reading a brief cover letter explaining the research protocol, patients elected to open the packet and complete the questionnaire or to return the packet without completing the questionnaire. Seventy percent of patients who were offered participation completed the study. Age and gender distributions were similar between participants and nonparticipants. Patients who completed the questionnaire were asked to consider sharing the results of the PHQ-9 portion with their physicians during their visit. 

### 2.2. Questionnaire

The survey instrument was four pages containing 38 large-print questions. Included were questions about demographics, religious beliefs, chronic medical problems, and a general rating of health status. Questions regarding depression included history of physician-diagnosed depression and previous treatments used. Using a five-point Likert scale, participants were asked about their beliefs regarding depression and the treatments that would be acceptable to them if they were depressed. The questionnaire was adapted from instruments in the literature, including the ADepT questionnaire [21]. The final portion of the questionnaire was the PHQ-9 diagnostic survey [22, 23].

### 2.3. Consent

 Capacity to consent was inferred by the participants' ability to travel to the clinic site and complete the necessary procedures to register into the clinic. If potential participants were unable to check into the clinic by themselves, their accompanying adult was given the research packet for consideration of having the elder patient participate. The project was approved by the University of Iowa Institutional Review Board.

### 2.4. Analysis

Descriptive statistics were obtained for all demographic and questionnaire variables. Mean differences in ratings of questionnaire items of ordinal variables were examined by *t*-test and one-way ANOVA. The chi-square test was used to examine the similarities in frequencies of categorical values. Subjects were grouped into depressed or non depressed categories by virtue of their answer to the question, “Do you have a history of depression?” PHQ-9 scores were used as a continuous variable to assess current depressive symptoms. 

Stepwise linear regression method was used to identify predictors of outcome variables. Each of the seven outcome variables was regressed on variables of demographics, attitudes about depression and treatments, and circumstances influencing treatment. Variables associated with an outcome variable with a *P* value of 0.20 or less were included as potential explanatory variables. In addition, the selected and excluded variables were checked for scientific plausibility based on past association with depressive symptoms. Using collinearity diagnostics for each final model, no strong collinearity was detected. The analysis was performed using SAS (SAS 9.2, SAS Institute, Inc., Cary, NC).

## 3. Results

The mean age of the study sample was 71.9 years; 59.4% were female, 57.1% married, and 65.7% retired. Less than 1% had no insurance, and 50% had a college or higher educational level. Self-reported health was listed as fair or poor by 19.2%, and 15% of the sample had four or more chronic diseases (Table 1).

Ninety-three (17.5%) participants had a past history of depression and were older (*P* = 0.05), had poorer self-reported health (*P* = 0.01), were more likely to be female (13% versus 5%, *P* = 0.005), and were likely to have had a diagnosis of heart disease or stroke (25% versus 16%, *P* = 0.04). Sixty (11.3%) had PHQ-9 scores of 10 or greater, and 134 (25%) had PHQ-9 scores of 5 or greater.

Participants believed depression is a disease, not a part of normal aging, and requires treatment. They perceived an inability to control depression by themselves and that treatment for depression is not embarrassing (Table 2). There was general agreement by participants that they could afford treatment, and they would not decline treatment because of age, life circumstances, or other more important medical problems. There was agreement that they would discuss treatment with their family and a sense that families would want them to be treated if they were depressed (Table 2). Participants also agreed that, if they were depressed, they would use medication and counseling and follow their doctor's recommendations. They were less likely to use prayer or herbal supplements and would not consider alcohol as a treatment option (Table 3).

Depressive symptoms measured by the PHQ-9 scores were associated with the degree of agreement with statements regarding depression and treatments (Table 4). Compared to nondepressed participants, participants currently with depressive symptoms were less likely to disagree with beliefs that “depression means you are weak,” “depression is a normal part of aging,” “treatment for depression is embarrassing,” “I could not afford treatment for depression,” and “I could not travel to receive treatment for depression.” Subjects with depressive symptoms were also less likely to agree to discuss treatment with family members.

In regression analyses, accepting medications from the primary physician was strongly associated with a past history of depression (*P* < 0.01) and with agreeing that depression needs treatment (*P* < 0.01) and that depression is caused by life events (*P* < 0.05). Agreement to receive medication from a psychiatrist was positively related to accepting the statement that depression needs treatment (*P* < 0.01) and negatively associated with the feeling that medications would not help (*P* < 0.001). Counseling was not acceptable treatment for those believing they can control depression on their own (*P* < 0.01) or those thinking treatment was embarrassing (*P* < 0.001). Older patients (*P* < 0.001) and those with higher education levels (*P* < 0.01) are less likely to accept herbs or supplements as treatment options. Persons with greater religious belief accept prayer as a treatment (*P* < 0.001) and would not use alcohol (*P* < 0.001). Willingness to discuss treatments with family was highly associated with not using alcohol and acceptance of all other treatment options (*P* < 0.001) (Table 5).

## 4. Discussion

Our sample exhibited a general attitude that depression is a disease physicians should ask about and is not necessarily a part of normal aging. Depression is thought to require treatment to improve, and being treated for depression is not embarrassing. These findings are similar to a nationally representative cross-sectional survey of American households showing positive mental health treatment beliefs in over 70% of persons 55 years of age or older [24]. Participants perceive that treatment is affordable, accessible and should not be avoided because of age, life circumstances, or other medical problems. These older adults would accept medication treatments for depression from their primary doctors or psychiatrists and consider counseling an acceptable intervention. This is consistent with a systematic review showing that between 49% and 84% of depressed or anxious patients perceive a need for counseling or medications [25]. As previously reported, most depressed older adults in primary care settings wish to receive some forms of treatment for their depression [26].

The types of treatments used by participants diagnosed with depression were not associated with the attitude variables. This may be due to a lack of power in the sample of 93 with past diagnoses of depression or may indicate, in persons accepting the diagnosis of depression, that attitudes do not influence treatment choices. Persons with greater depressive symptoms tended to agree or disagree in the same direction regarding the attitudes about depression as older persons without depressive symptoms. The degree of agreement, however, differed for certain statements. Persons without depressive symptoms strongly disagreed with the statements that having depression means that the person is weak and that treatment is embarrassing, whereas those with depressive symptoms only disagreed. A striking finding was in response to the willingness to discuss treatment with family members. Greater depressive symptoms were associated with less conviction that a person would discuss treatment with family. If physicians believe that it is beneficial to have family members involved in caring for depressed patients, it may be wise to encourage patients to have a family member accompany them to office visits and not to rely on patients to discuss depressive symptoms and treatment compliance issues through their own initiative. 

Those who believed that depression is caused by life events had greater acceptance of antidepressants for treatment of their mood. Persons who feel that they have little intrinsic ability to impact their mood or environment are defined as having low internal locus of control and, conversely, a high external locus of control. Previous research has demonstrated an independent link between high external locus of control and depression. Our observation may reflect a desire among those with low internal locus of control to seek out treatment that depends upon as little internal change as possible [27]. Persons believing that they can control depression on their own have reduced acceptance of counseling and a greater tendency to rely on herbal supplements. Herbal supplements can be purchased without prescriptions or need to interact with a physician, enhancing the person's ability to self-manage their depression. 

Perceptions regarding depression from older adults who are not depressed are also important because of their risk of developing depression later in life related to cardiovascular and cerebrovascular disease or other chronic diseases. This group may also have spouses suffering from depression, and their attitudes may impact the spouses' treatment. Caregivers' beliefs about the root of patient's depression can strongly influence medication adherence [28]. In a study examining adherence to lithium therapy for affective disorders, marriage was correlated with better adherence over a one-year period [29]. Marital status had a strong relationship with adherence to citalopram treatment in adults aged 60 years and older being seen in a primary care setting [30]. Additionally, those with poor marital support were more likely to have recurrent depressive symptoms and fail to comply with treatment one year after optimum medical therapy [31].

A striking correlate was found regarding the willingness to discuss treatment with family members. Greater depressive symptoms were associated with less conviction that a person would discuss treatment with family; however, there was a strong and consistent correlation with discussion of depression with family and willingness to accept all treatment options after accounting for all other variables. Paradoxically, those most in need of pursing treatment options for their mood may be the very people least willing to discuss treatment with their loved ones, leading to poorer outcomes and undue suffering. This finding is consistent with the use of psychoeducational workshops for depressed patients and their families to promote continuation treatment for the depressed patients [32]. The willingness to discuss treatment options with family is a condition associated with enhanced acceptance of medication use, counseling, and following the doctor's recommendations. Encouraging patients to have a family member accompany them to office visits is one approach to enhance family communications regarding the importance of symptom monitoring and treatment compliance.

 Our sample was insured, lived in an area with accessible mental health resources, and did not report cost as a barrier to depression treatment. The most common reason for not seeking mental health treatment in a sample of 6,510 adults was concern about costs [33]. Significant proportions (19% to 38%) of elderly subjects with depressive symptoms report cost-related nonadherence to medications [34]. Differing healthcare plans have been linked to poor mental health followup [35] and have been shown to impact access to antidepressant medication management and other treatment options [36]. Counseling services are highly variable among private and state-run health insurances and dependent on local access and availability of qualified mental health professionals, which may also explain some of the variation in our results locally and on a national level. Further research is needed to assess the impact of insurance coverage on the acceptance of treatments for depression. 

Several potential clinical implications arise from this exploratory study. Screening for depression in primary care settings is acceptable to older patients. Attitudes of patients and their circumstances may help predict the acceptability of and compliance with depression treatments. Attitudes should be queried after making the diagnosis and before prescribing treatments. This is similar to the recommendation made following the study of younger depressed primary care patients in the United Kingdom [21]. Family involvement in treatment is an enhancing factor in treatment acceptability. Offering to discuss depression and treatment options with both patient and family may help improve compliance and treatment outcomes.

There are several limitations to the study, including generalizability and power to detect significant findings in the smaller depressed sample. The study sample was mainly drawn from an academic primary care setting and may not represent the general older, ambulatory population. Although 50 percent of the sample had a college education and less than one percent had no insurance, characteristics such as number and kinds of chronic diseases, general health ratings, and other demographic variables were similar to the general older population. 

To overcome these limitations, a similar study needs to be conducted with a larger sample size, preferably in a primary care research network. If attitudinal factors are related to treatment acceptability, a screening of attitudes towards depression should be used in an intervention trial. Physicians do inquire about the acceptability of treatments before prescribing, but better understanding of the patients' and families' beliefs may help determine the extent of education needed for any individual patient. If a strong relationship between family involvement and treatment acceptance is confirmed, a comparison trial between usual care and a family intervention trial would be of value, with the outcome variables of remission of depression and compliance with continuation treatment. 

## 5. Conclusions

It is important to understand the patients' perceptions regarding depression, as well as other enhancers or barriers to effective treatment. Patients believe that depression is a disease that requires treatment and that physicians should ask their patients about depressive symptoms. The willingness to discuss depression with family appears to be associated with greater acceptance of depression treatments. Physicians' facilitation of these family discussions may be an effective approach to increase treatment compliance and improve depression outcomes.

## Figures and Tables

**Table 1:** Characteristics of the study sample *N* = 529, (  ) = %.

Age	
Mean	71.9 yrs
Range	60–93 yrs
Gender	
Males	(40.6)
Females	(59.4)
Marital status	
Married	(57.1)
Widowed	(20.2)
Divorced	(15.7)
Single, never married	(5.7)
Other	(1.3)
Employment status	
Retired	(65.7)
Employed	(21.9)
Disabled	(6.1)
Homemaker	(4.8)
Unemployed	(1.5)
Education	
<12 yrs	(5.7)
12–15 yrs	(44.4)
≥16 yrs	(49.9)
Medical visits past 3 months	
None	(21.9)
1–3	(58.7)
4–6	(13.7)
>6	(5.7)
Medical insurance*	
Medicare	(79.1)
Private	(58.9)
Medicaid	(12.6)
Other	(17.2)
None	(0.6)
Religious beliefs	
Strong	(36.5)
Average	(41.7)
Weak	(10.6)
None	(11.2)
Religious services attended	
>1/week	(8.8)
1/week	(30.6)
1-2/month	(13.3)
1-2/year	(13.6)
Almost never	(33.7)
Number of chronic diseases^+^	
0	(17.4)
1	(27.3)
2	(26.7)
3	(13.5)
≥4	(15.1)
General health rating	
Excellent	(8.1)
Very good	(31.0)
Good	(41.7)
Fair	(16.9)
Poor	(2.3)

*More than one answer may apply.

^+^Disease prevalence: arthritis (45.0), hypertension (42.4), diabetes (19.1), thyroid disease (17.6), nonskin cancer (13.5), asthma/bronchitis (11.8), heart attack (8.4), heart failure (6.3), angina (6.3), and stroke (5.9).

**Table 2:** Attitudes about depression and treatment.

	*N*	Mean*	Std. dev.
Depression			
Depression is a kind of disease	492	4.21	1.076
Depression is a personality trait	471	2.62	1.264
Having depression means that the person is weak	480	1.51	.993
Physicians should ask patients about depression	483	4.39	1.031
Depression is a normal part of aging	483	2.49	1.265
Depression is caused by one's life events	482	3.63	1.124
Treatment			
Depression needs to be treated	500	4.69	.786
Depression improves without treatment	479	2.40	1.222
Treatment for depression is embarrassing	459	1.82	1.210
Taking medication will make me feel sick	457	2.00	1.168
I can control depression by myself	458	2.41	1.374
Medication for depression would not help me	462	1.83	1.059
Circumstances influencing treatment			
I could not afford treatment	454	2.11	1.294
I would not be able to travel to the hospital or clinic to receive treatment	451	1.69	1.173
My family would not agree to have me treated	456	1.42	.908
Because of my age, I would not want treatment	456	1.47	.970
Because of my life circumstances, I would not want treatment	459	1.50	1.012
Because of many other more important medical problems, I would not want treatment	453	1.50	.981
I would discuss treatment with my family	455	3.95	1.383

*Scales 1–5, where 1 means strongly disagree, 2 means somewhat disagree, 3 means do not know, 4 means somewhat agree, and 5 means strongly agree.

**Table 3:** Treatment options.

Statements: if I were depressed, I would	*N *	Mean*	Std. dev.
Take medication prescribed by my primary physician	466	4.35	1.145
Take medication prescribed by my psychiatrist	455	4.30	1.134
Go for counseling	462	4.26	1.135
Follow my doctor's recommendations	460	4.48	.989
Use prayer and meditation as a treatment	460	3.19	1.517
Take an herb or other supplement	447	2.63	1.414
Increase my alcohol intake	455	1.28	.773

*Scales 1–5, where 1 means strongly disagree, 2 means somewhat disagree, 3 means do not know, 4 means somewhat agree, and 5 means strongly agree.

**Table 4:** Depressive symptoms and associations with attitudes about depression and treatment.

Statement	PHQ-9 score	*N *	Mean*	Std. dev	*F* ^†^	*P* value
Depression is a kind of disease	0–4	348	4.27	1.047	3.052	.048
	5–14	107	4.03	1.169		
	15 & up	26	4.50	.583		

Having depression means that the person is weak	0–4	338	1.46	.924	6.094	.002
	5–14	108	1.54	1.027		
	15 & up	26	2.15	1.488		

Depression is part of normal aging	0–4	340	2.39	1.240	5.226	.006
	5–14	108	2.79	1.261		
	15 & up	25	2.88	1.301		

Treatment for depression is embarrassing	0–4	325	1.73	1.118	5.013	.007
	5–14	103	2.09	1.380		
	15 & up	24	2.25	1.511		

I could not afford treatment	0–4	324	1.98	1.202	7.288	.001
	5–14	100	2.47	1.473		
	15 & up	24	2.58	1.442		

I would not be able to travel to the hospital or clinic to receive treatment	0–4	321	1.58	1.073	6.031	.003
	5–14	101	1.95	1.374		
	15 & up	23	2.17	1.403		

Because of my age, I would not want treatment	0–4	323	1.40	.866	3.521	.030
	5–14	102	1.66	1.130		
	15 & up	24	1.71	1.367		

Because of my life circumstances, I would not want treatment	0–4	325	1.43	.926	3.852	.022
	5–14	103	1.75	1.226		
	15 & up	24	1.54	1.103		

Due to many other more important medical problems, I would not want treatment	0–4	322	1.43	.918	4.431	.012
	5–14	102	1.75	1.103		
	15 & up	23	1.57	1.199		

I would discuss treatment with my family	0–4	323	4.05	1.361	5.085	.007
	5–14	103	3.85	1.339		
	15 & up	24	3.17	1.579		

I would take medication prescribed by my psychiatrist	0–4	324	4.31	1.142	3.208	.041
	5–14	102	4.15	1.189		
	15 & up	24	4.79	.588		

*Scales 1–5, where 1 means strongly disagree, 2 means somewhat disagree, 3 means do not know, 4 means somewhat agree, and 5 means strongly agree.

^†^ANOVA.

**Table 5:** Multiple linear regression analyses on the treatment options.

Predictors	Regression coefficient	*t* value	*P* value
Meds from primary physician (*R* ^2^ = 0.16)			
(i) History of depression	.34	2.64	.0088
(ii) Depression needs to be treated	.22	2.61	.0093
(iii) Depression is caused by one's life events	.11	2.08	.0381
(iv) Discuss treatment with family	.18	4.18	<.0001
(v) Depression improves without treatment	−.11	−2.26	.0247
(vi) Medications for depression would not help	−.13	−2.17	.0310
Meds from psychiatrist (*R* ^2^ = 0.24)			
(i) Higher education	.22	2.25	.0249
(ii) Depression needs to be treated	.22	2.65	.0084
(iii) Depression is caused by one's life events	.12	2.36	.0188
(iv) Discuss treatment with family	.19	4.63	<.0001
(v) Depression is a personality trait	−.09	−2.05	.0413
(vi) Medications for depression would not help	−.27	−4.68	<.0001
Counseling (*R* ^2^ = 0.23)			
(i) Higher education	.26	2.83	.0049
(ii) Family does not agree with treatment	.16	2.44	.0152
(iii) Discuss treatment with family	.21	5.45	<.0001
(iv) Treatment for depression is embarrassing	−.17	−3.60	.0004
(v) I can control depression by myself	−.13	−2.91	.0039
(vi) Medications for depression would not help	−.17	−2.94	.0035
Follow MD's recommendations (*R* ^2^ = 0.18)			
(i) Higher education	.26	3.08	.0023
(ii) Discuss treatment with family	.20	5.73	<.0001
(iii) Meds for depression would not help	−.22	−4.59	<.0001
Prayer and meditation (*R* ^2^ = 0.29)			
(i) Religious belief	.76	11.38	<.0001
(ii) Discuss treatment with family	.18	3.80	.0002
(iii) Pay health expenses with my earnings	−.37	−2.63	.0088
Use herbs or supplements (*R* ^2^ = 0.10)			
(i) Can control depression by myself	.19	3.46	.0006
(ii) Discuss treatment with family	.14	2.73	.0066
(iii) Older age	−.03	−3.48	.0006
(iv) Higher education	−.37	−3.01	.0028
Increase alcohol intake (*R* ^2^ = 0.14)			
(i) Due to life circumstances, I do not want treatment	.11	2.45	.0149
(ii) Medications for depression would not help	.09	2.12	.0347
(iii) Discuss treatment with family	−.10	−3.41	.0007
(iv) Pay health expenses from my earnings	−.21	−2.62	.0092
(v) Religious belief	−.14	−3.43	.0007
